# Galactocele in a prepubertal girl: case report

**DOI:** 10.1093/jscr/rjaf593

**Published:** 2025-08-06

**Authors:** Souha Qarouach, Monim Ochan, Hadjar Nassiri, Jaouad Bouljrouf, Mounir Kisra

**Affiliations:** Université Mohammed V de Rabat, Faculté de medecine et de pharmacie de Rabat, Av. Mohamed Belarbi El Alaoui, B.P. 6203, 10000 Rabat, Morocco; Ibn Sina University Hospital Center, Pediatric Surgery, Rue Lamfadel Cherkaoui, B.P. 6527, 10000 Rabat-Sale-Zemmour-Zaer, Rabat, Morocco; Université Mohammed V de Rabat, Faculté de medecine et de pharmacie de Rabat, Av. Mohamed Belarbi El Alaoui, B.P. 6203, 10000 Rabat, Morocco; Ibn Sina University Hospital Center, Pediatric Surgery, Rue Lamfadel Cherkaoui, B.P. 6527, 10000 Rabat-Sale-Zemmour-Zaer, Rabat, Morocco; Université Mohammed V de Rabat, Faculté de medecine et de pharmacie de Rabat, Av. Mohamed Belarbi El Alaoui, B.P. 6203, 10000 Rabat, Morocco; Ibn Sina University Hospital Center, Pediatric Surgery, Rue Lamfadel Cherkaoui, B.P. 6527, 10000 Rabat-Sale-Zemmour-Zaer, Rabat, Morocco; Abdelmalek Essaadi University, Laboratory of Life and Health Sciences, Faculty of Medicine and Pharmacy of Tangier, Route du Charf, B.P. 1255 Tanger Principale 90000, Tangier-Tetouan, Tetouan, Morocco; Université Mohammed V de Rabat, Faculté de medecine et de pharmacie de Rabat, Av. Mohamed Belarbi El Alaoui, B.P. 6203, 10000 Rabat, Morocco; Ibn Sina University Hospital Center, Pediatric Surgery, Rue Lamfadel Cherkaoui, B.P. 6527, 10000 Rabat-Sale-Zemmour-Zaer, Rabat, Morocco; Université Mohammed V de Rabat, Faculté de medecine et de pharmacie de Rabat, Av. Mohamed Belarbi El Alaoui, B.P. 6203, 10000 Rabat, Morocco

**Keywords:** galactocele, male, girl, infant

## Abstract

Galactocele is a rare, benign cystic breast lesion that usually occurs in breastfeeding women, less frequently in children, and more frequently in boys. We report a rare galactocele case in a 2-year-old girl with a painless unilateral breast mass. Imaging was consistent with a cystic lesion, and surgical excision confirmed the diagnosis. Galactocele in this case runs contrary to the male predominance reported in the literature. We discuss the importance of galactocele in the differential diagnosis of breast masses and emphasize its management with monitoring, aspiration, or surgical excision. The etiology remains unknown although it seems to be multifactorial.

## Introduction

A galactocele is a rare benign breast lesion that generally develops during or after lactation in women and affects exclusively infants and children. Interestingly, in the cases previously reported in the global literature, the majority of subjects were male [1, 2]. These cysts can present as painless unilateral or bilateral masses [3]. We report a case of galactocele in a 2-year-old girl.

## Case report

We report the case of a 2-year-old girl who was referred to the pediatric surgery department for a unilateral left breast mass, evolving since the age of 9 months. She had no significant medical history. The mass gradually increased in size without any signs of pain or tenderness. No galactorrhea was present.

**Figure 1:**
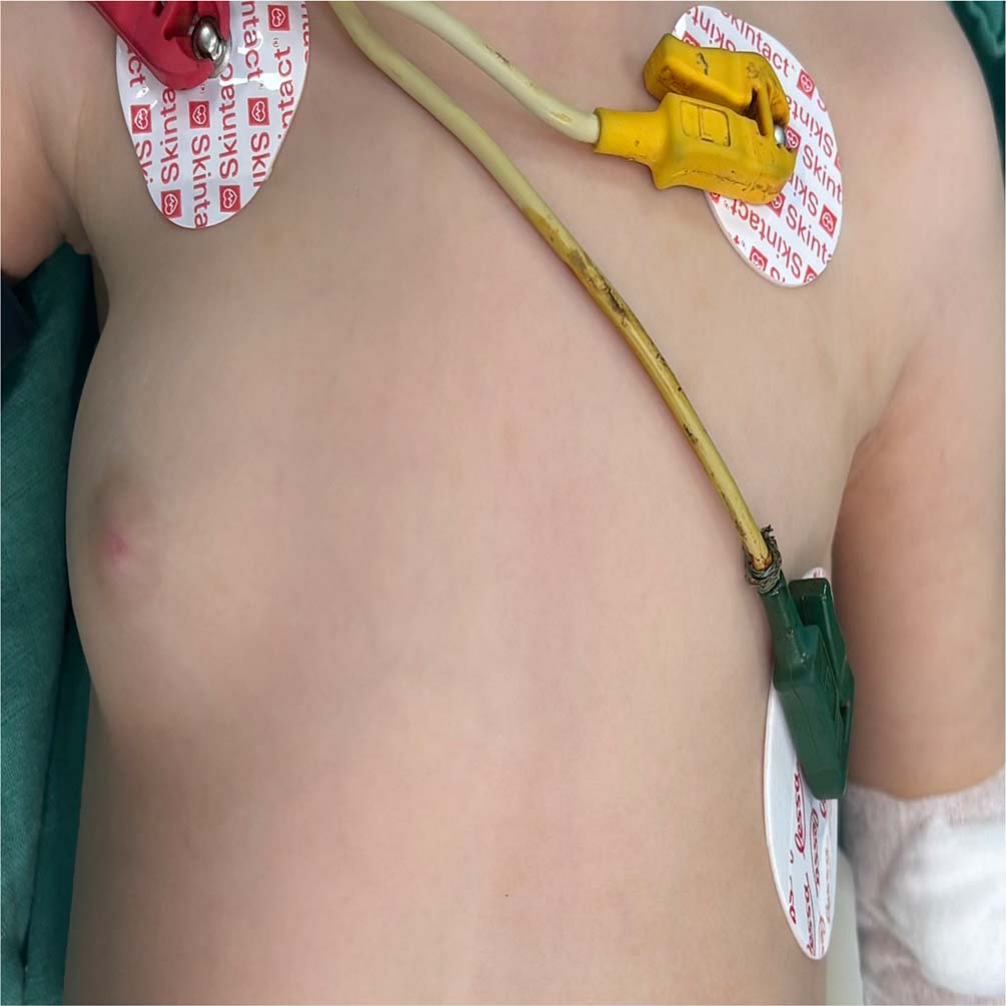
Right breast tumor of a female 2-year-old breastfed infant; tumor growth occurred since the child was 9 months old.

**Figure 2:**
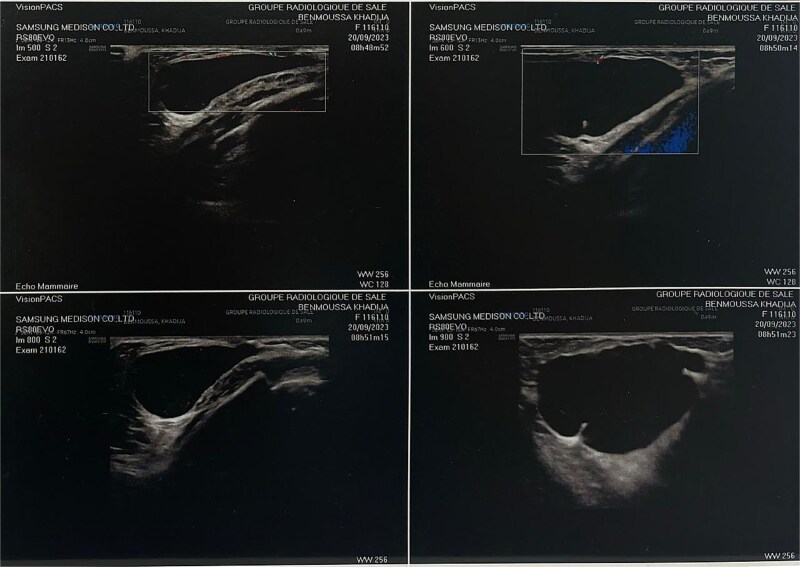
Ultrasonography of left breast showing a cystic lesion.

On physical examination, a soft and spongy mass was palpable in the left breast (Fig. 1). A breast ultrasound was performed, revealing an oblong, well-defined cystic mass with regular contours, an anechoic content containing fine mobile echogenic echoes, and a few thin, incomplete septa, measuring 37 × 46 mm (Fig. 2). The ultrasound concluded to a cystic breast mass, most likely a galactocele. A hormonal workup including prolactin, luteinising hormone, follicle-stimulating hormone, and estradiol was performed, with all values within the normal range.

**Figure 3:**
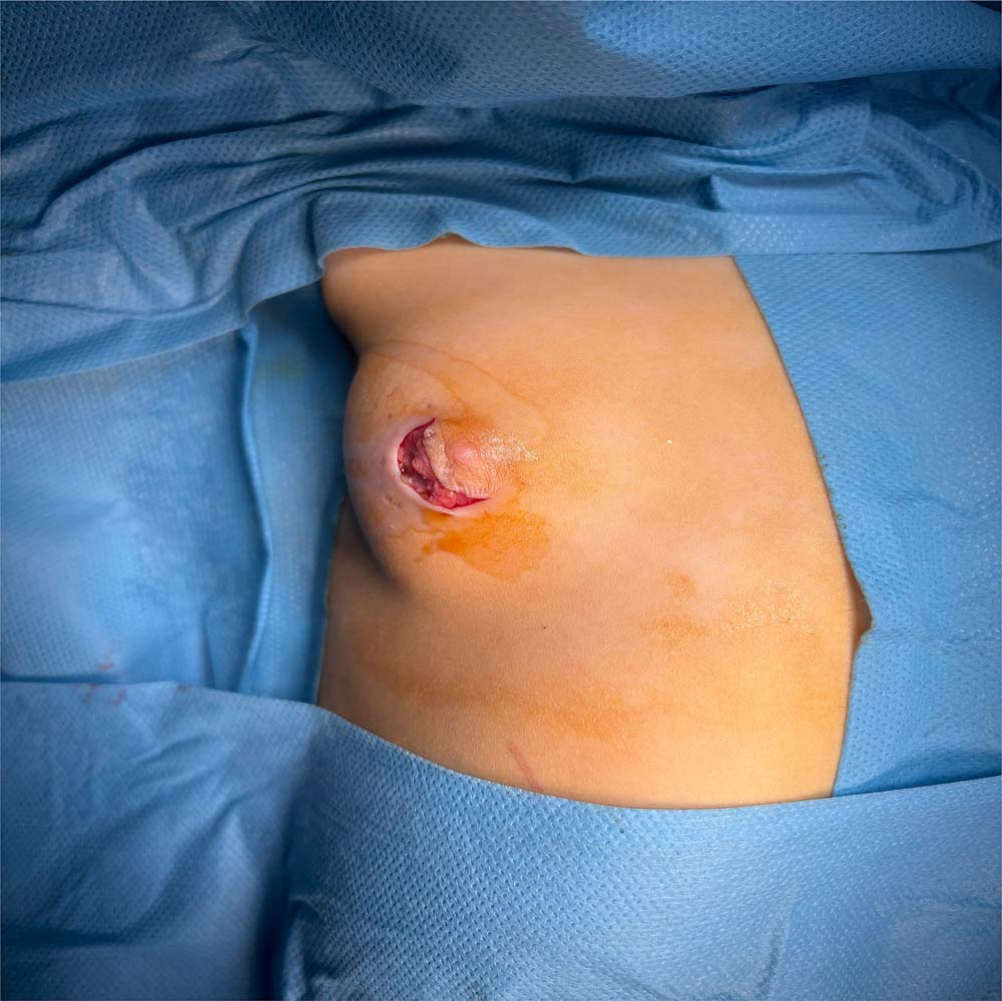
Preoperative image showing the incision.

**Figure 4:**
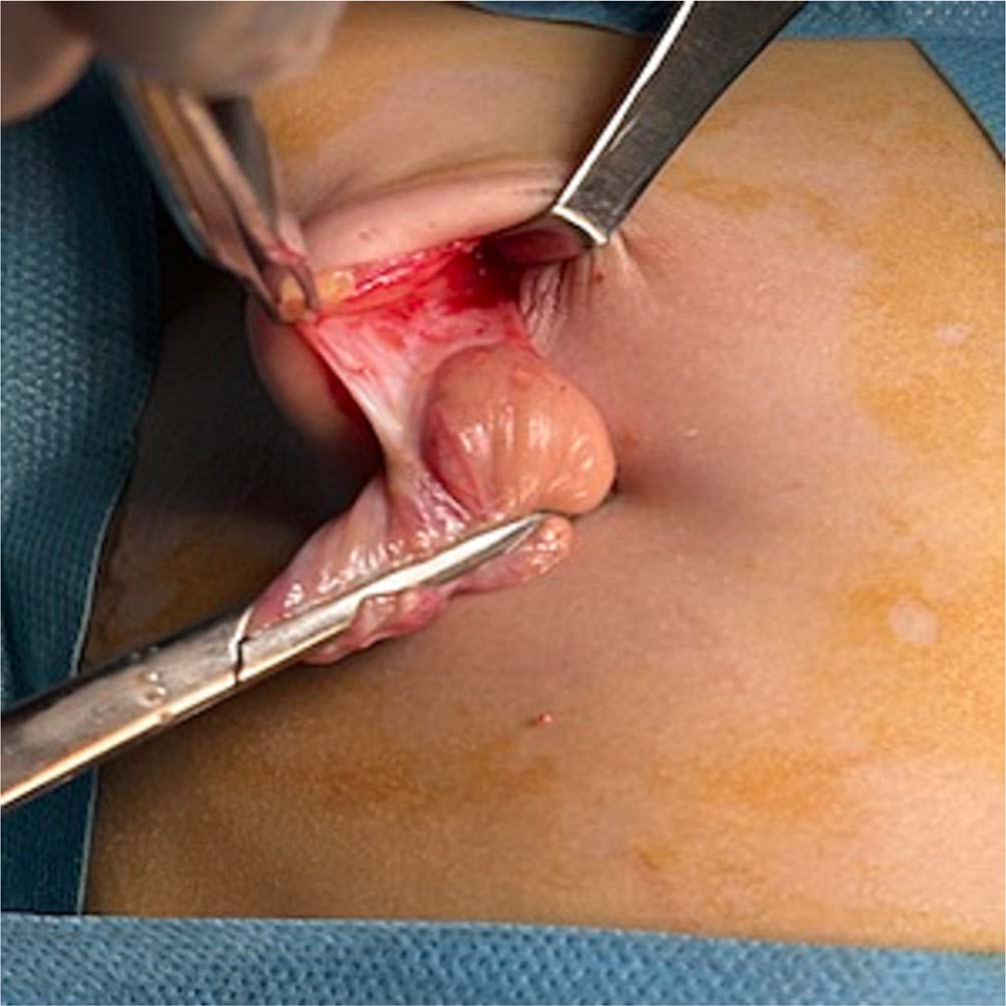
Image of the cyst in preoperative.

**Figure 5:**
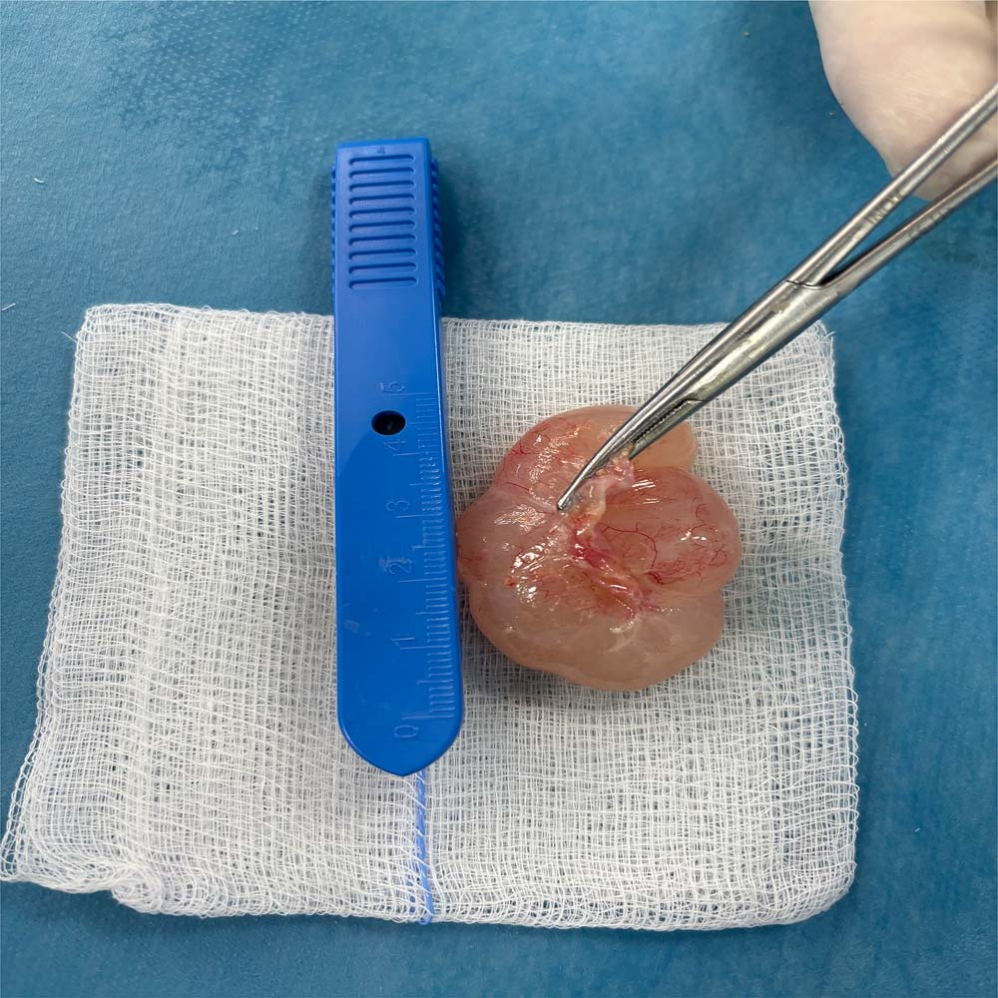
Image showing the resected mass.

**Figure 6:**
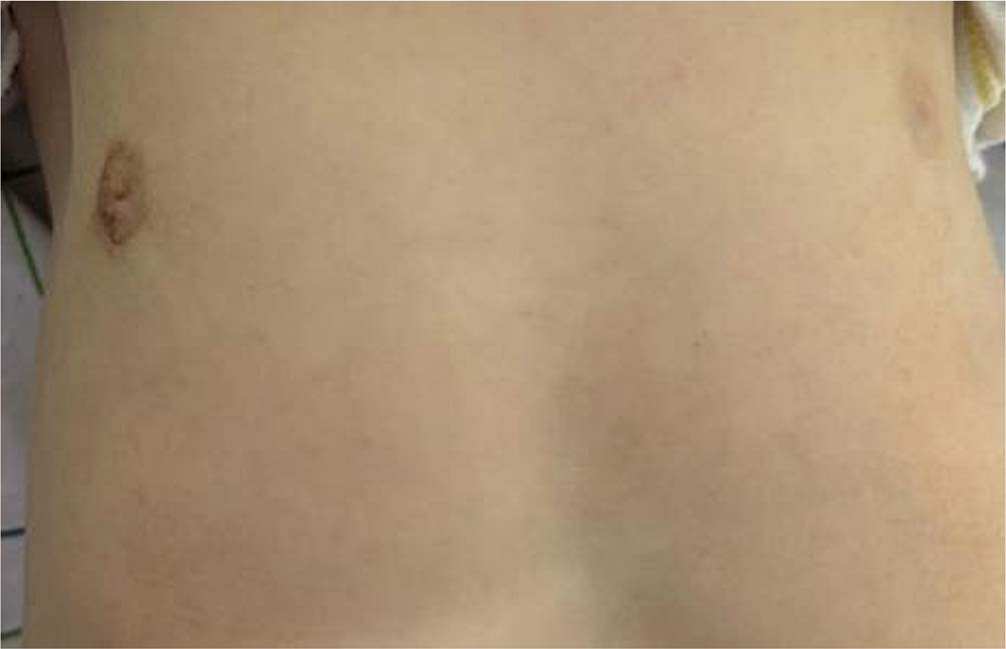
Image of the patient at 2 months post-operative.

Surgical exploration of the right breast was performed through a lateral external arc-shaped incision (Fig. 3), and a cystic mass (Fig. 4) containing opalescent fluid was removed (Fig. 5). Histopathological examination of the mass confirmed the diagnosis of galactocele. At 2-month follow-up, the wound had healed well with no evidence of recurrence (Fig. 6).

## Discussion

Galactoceles are thin-walled cysts filled with milk, most commonly seen in lactating women of childbearing age and usually linked to the obstruction of a galactophorous duct. Spontaneous resolution is rare, and either aspiration or surgical excision is needed when the cyst is troublesome [4].

Galactocele has also been described in male children as painless, unilateral or bilateral breast enlargement without associated hormonal abnormalities [1].

Several authors have identified three main factors in the background of a galactocele: past or present stimulation by prolactin, secretion from mammary epithelial cells with cystic retention due to a trauma and subsequent inflammatory reaction, ductal obstruction with absence of canaliculus formation resulting in fluid collection, and formation of a cyst [5, 6].

Other studies suggest that galactoceles result from small retention cysts formed during the neonatal period, which remain asymptomatic until an inflammatory reaction is induced by trauma [1–7].

In cases of cystic breast lesions in children, the differential diagnosis is lymphatic malformation, which is a very common disease. Ultrasound diagnosis is not always easy, as interpreting the images and differentiating between pathologies can be challenging, especially since young patients often have difficulty remaining still during the examination. Magnetic resonance imaging, which offers a better soft tissue resolution and is less dependent on the operator, has been used in only four published cases [8–10].

Treatment consists of either monitoring the galactocele with serial clinical or ultrasound examinations or aspirating the galactocele to provide symptomatic relief [11].

Within the published literature, infra-areolar excision of the cyst was the chosen management in 23 out of 26 reported cases of galactocele in boys (88%) [8].

Although it is well known that galactoceles can occur in children and adolescents, the few publications available do not explain why galactoceles occur in male infants. This rare disease will probably be the subject of future publications, and in our opinion, the disease is probably multifactorial.

## Conclusion

Galactocele is a rare but important differential diagnosis of breast masses in children, classically described in male infants. This case further illustrates the occurrence of this entity in a female child and highlights that clinicians should consider this entity regardless of sex. Imaging is the key to diagnosis, with histopathological confirmation useful. Management should be tailored to the individual presentation, from observation to surgical excision. Given the rarity and uncertain pathogenesis of pediatric galactoceles further studies are required to better understand this entity and optimal management.
